# A Prospective, Randomized, Double‐Blind, Split‐Face Trial Evaluating the Effects of Pre‐Conditioning With a TriHex Technology Serum Nectar 2.0 Versus a Vehicle in Subjects Undergoing Elective Facelift Surgery

**DOI:** 10.1111/jocd.70599

**Published:** 2025-12-08

**Authors:** Alan D. Widgerow, Amir Moradi, Reza Sadrian, Kiersten Riedler, Faiza Shafiq, Tiffany Robison

**Affiliations:** ^1^ Division Chief Research, Professor Plastic Surgery, Center for Tissue Engineering University of California Irvine California USA; ^2^ Pacific Clinical Innovations Vista California USA; ^3^ Sadrian Plastic Surgery La Jolla California USA; ^4^ La Jolla Cosmetic Surgery Centre La Jolla California USA; ^5^ Alastin Skincare, Inc., a Galderma Company Carlsbad California USA

## Abstract

**Background:**

Preconditioning of skin prior to procedures with the Alastin TriHex Technology has been demonstrated to enhance procedure outcomes. Alastin Regenerating Skin Nectar with TriHex Technology Serum (Alastin Skincare Inc. Carlsbad, CA) has undergone reformulation to include an added Octapeptide‐45 for improving post‐surgical outcomes and healing through pre‐conditioning. A 5‐week clinical trial was conducted to demonstrate efficacy and safety for post‐surgical skin wound healing (following pre‐conditioning), and overall skin health. In particular, molecular and cellular changes were documented, histologically comparing the treated side against the vehicle on the opposite side.

**Methods:**

A randomized, double‐blinded, split‐face, comparative study was conducted in 5 patients to evaluate the effects of pre‐conditioning with a TriHex Technology Serum 2.0 (Nectar 2.0) compared to vehicle in subjects undergoing elective facelift surgery. Patient questionnaires and investigator assessments were undertaken, and baseline biopsies were taken from both sides 4 weeks prior to surgery. Following the facelift surgery, collected skin samples from each side of the face were analyzed for histological changes comparing pre (screening) and post (4 weeks) of pre‐conditioning use of study topicals by an independent dermatopathologist using multiple staining techniques.

**Results:**

Investigator assessment with facial aesthetic clinical grading scale demonstrated greater improvements for the Nectar 2.0 treatment side compared to vehicle at Day 7 from screening for all assessed parameters: skin dullness, fine lines, tactile roughness, global photodamage. Investigator healing assessments demonstrated improved Healing on the Nectar 2.0 treatment side compared to vehicle, related to erythema, edema, and crusting parameters. Histological differences between the two sides were significant with major changes in extracellular matrix composition involving collagen, elastin and hyaluronic acid production and reversal of solar elastosis, all Evident only on the Nectar 2.0 treated side.

**Conclusion:**

A randomized, double‐blinded, split‐face, comparative study with Alastin Regenerating Skin Nectar with TriHex + Technology Serum 2.0 revealed that pre‐conditioning resulted in significant improvement in healing, global skin health and histological results with marked remodeling of the ECM being observed in all patients. A short period of pre‐conditioning can have substantial effects on skin health and procedure outcomes.

## 1 Introduction

Preconditioning of the skin prior to procedures with the Alastin TriHex Technology (Alastin Skincare Inc., Carlsbad, CA) has been demonstrated to enhance procedure outcomes prior to surgical procedures [1]. This concept of “skin bed preparation” prior to surgical procedures was adapted from chronic wound bed preparation, a concept that aimed at changing the wound milieu decreasing waste products and inflammatory components prior to applying any therapeutic [2]. Similarly, “skin bed preparation” targets the extracellular matrix (ECM), removing fragmented collagen and elastin, remodeling the matrix to create a regenerative environment for promoting optimal skin health and healing outcomes [1]. This study advances the preconditioning concept by introducing a reformulated Alastin Regenerating Skin Nectar with TriHex + Technology Serum 2.0 (Nectar 2.0) with the addition of an Octapeptide‐45 to the TriHex Technology creating a combination that has proven additive effects on the ECM, dermo‐epidermal junction and fibroblast senescence [3]. The synergy between TriHex Technology and the added Octapeptide‐45, now referred to as TriHex + or Next Generation TriHex Technology Serum 2.0, has been thoroughly validated through gene expression, and multiple in vitro and ex vivo studies and once again demonstrates the power of peptide technology [3]. As we did with the first generation TriHex Technology, we now explore the molecular changes that are observed through this process of pre‐conditioning in vivo. In particular, we demonstrate powerful ECM remodeling with added regenerative capacity, once again confirming that a short period of preparation prior to a procedure can go a long way in changing the molecular milieu and hence the regenerative potential of the treated area.

## 2 Material and Methods

### 2.1 Facelift Surgery and Histology

A randomized, double‐blinded, split‐face, comparative study was conducted to evaluate the effects of pre‐conditioning with Nectar 2.0 for improving overall skin health compared to Vehicle in subjects undergoing elective facelift surgery from Sep 2023 to Dec 2024. The study was approved by Veritas IRB Inc. (Montreal, Quebec, Canada) and all subjects were consented prior to any study procedures being completed. Study participants were scheduled for elective face‐lift surgery at the investigative site 4 weeks from study enrollment. Subjects then completed questionnaires, underwent Investigator assessments, and 3 mm punch biopsy collections from the pre‐auricular area of each side of the face at screening. Subjects were then randomized to a split‐face regimen of Nectar 2.0 and Vehicle on the assigned left or right side of the face. Subjects then initiated use of the skincare regimens to the assigned side of the face for 4 weeks as a preconditioning phase prior to baseline/facelift surgery day (Day −28). Supporting skincare regimen products included a cleanser (Ultra Calm Cleansing Cream, Alastin Skincare Inc., Carlsbad, CA), and sunscreen (SilkSHIELD SPF 30, Alastin Skincare Inc., Carlsbad, CA or HydraTint Pro Mineral Broad Spectrum Sunscreen SPF 36, Alastin Skincare Inc., Carlsbad, CA). On the day of the facelift procedure (Day 0), subjects completed questionnaires and Investigators performed study assessments. Following the facelift surgery, collected skin samples from each side of the face were placed in separate containers and underwent evaluation, along with the biopsy samples, for histological changes pre (screening) and post (4 weeks) of pre‐conditioning use of study topicals by an independent dermatopathologist using the following stains: Movat, Herovici, H&E (Hematoxylin and Eosin), and CD44.

The investigator instructed subjects according to their standard of care when to resume application of the assigned study skincare regimen products. Subjects continued applying the assigned skincare regimens daily until the final study visit. Post procedure, subjects returned to the clinical site for follow‐up on Days 2, 5, and 7 for evaluation. At each follow‐up visit, subjects completed questionnaires, and investigator assessments were performed. Each participant was involved in the study for up to 5 weeks, including a 28‐day preconditioning phase. Efficacy and safety data were monitored and collected at each study visit.

Statistical analyses were performed using descriptive statistics to compare changes from screening and baseline to the end of study Day 7. The percentage and frequency of response were tabulated for the Subject Product Questionnaires. A higher percentage of favorable responses was used to determine product superiority and subject preference. In addition to multiple specialized stains, biopsy image analysis was performed to quantify changes in CD44 expression pre and post use of the study topicals using ImageJ software [3].

### 2.2 Assessments

#### 2.2.1 Investigator Assessments

Clinical Grading was performed by Investigators using a 10‐point Modified Griffith's Scale (0 = None, 1–3 = Mild, 4–6 = Moderate, and 7–9 = Severe) to assess facial skin parameters on each side (left and right) individually (global photodamage, fine wrinkles, skin dullness, tactile roughness). Subjects were instructed to maintain a relaxed facial position during the assessment.

Investigator Healing Assessments were performed to evaluate the surgical incision sites on both sides of the face post facelift for safety healing parameters (erythema, edema, crusting, and exudation) using a 4‐point scale (0 = None, 1 = Mild, 2 = Moderate, 3 = Severe).

#### 2.2.2 Subject Assessments and Questionnaires

Subjects completed the Subject Self‐Assessment Questionnaire at the Screening Pre‐Conditioning Phase visit (1b) prior to product use, at Baseline (Day 0) prior to facelift surgery, and at Visit 5 EOS to self‐assess their current skin perception EOS by selecting 1 of 5 responses (Strongly Agree, Agree, Neither Agree Nor Disagree, Disagree, Strongly Disagree), for each of the following statements for both the right and left sides of the face: (1) My skin feels smooth, (2) My skin appears bright, (3) My skin looks healthy.

Subject tolerability was assessed at home on the day of the Screening Pre‐Conditioning Phase visit (1b), where participants completed a Subject Tolerability Assessment to grade the degree of burning, itching, or stinging/tingling experienced post study topical application on each treatment side using a 4‐point scale (0 = None, 1 = Mild, 2 = Moderate, 3 = Severe).

Subjects reported their perception and satisfaction with the study topical treatments and skincare regimen products by completing the Subject Product Assessment Questionnaire upon initiating the preconditioning phase at home post screening visit, visit 2 prior to facelift, and visit 5 EOS, by selecting 1 of 5 responses (Strongly Agree, Agree, Neither Agree Nor Disagree, Disagree, Strongly Disagree), to each of the following statements: (1) This product spreads easily, a little goes a long way, (2) I feel that the product improves my skin hydration, (3) My skin looks recovered, (4) The product conditions my skin, (5) The product soothes my skin, (6) I feel the product does not irritate my skin, (7) The product helps to improve the overall appearance of my skin, (8) My skin looks firmer, (9) My skin looks younger, rejuvenated, (10) I feel the product helps to improve my skin texture, (11) The appearance of fine lines/wrinkles looks reduced, (12) Make‐up applies over the product better, (13) This is a game‐changing product.

Subjects also completed an end of study survey by selecting 1 of 5 responses (Strongly Agree, Agree, Neither Agree Nor Disagree, Disagree, Strongly Disagree), for each of the following statements for both the right and left sides of the face: The Test Product did not cause facial skin (1) dryness, (2) irritation, (3) redness, (4) overall satisfaction. Continued Test Product use and recommendation also were assessed for both sides of the face by responding either yes or no.

## 3 Results

Overall, 6 subjects were enrolled in the study, and 5 completed, as 1 subject early terminated due to facelift surgery cancellation. Mean age for completed subjects was 58.4 years (Range: 55–63 years); 100% (*n* = 5) were female. Study Subject Fitzpatrick skin type distribution included, 40% (*n* = 2) were Type II, 40% (*n* = 2) were Type III, and 20% (*n* = 1) were Type IV.

### 3.1 Assessments

#### 3.1.1 Investigator Assessments

**FIGURE 1:**
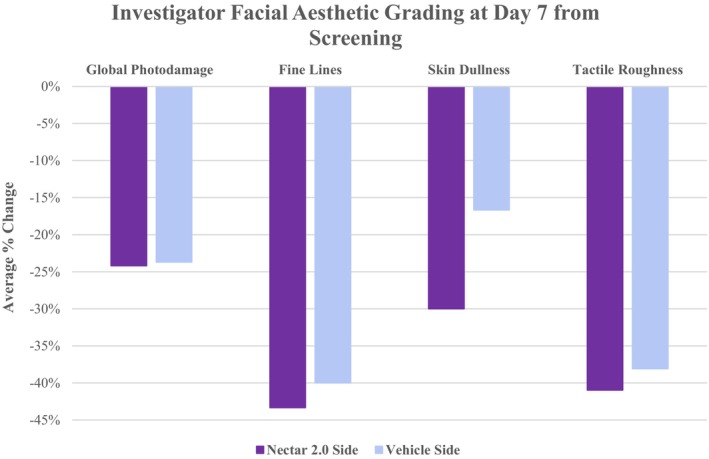
Average % Change at Day 7 from Screening, where Nectar 2.0 achieved greater improvements (decreased) in severity ratings compared to Vehicle.

Facial Aesthetic Clinical Grading performed using a 10‐point Modified Griffith's Scale demonstrated greater improvements on average for the Nectar 2.0 treatment side compared to Vehicle at Day 7 from screening for all assessed parameters: skin dullness (13.33%), fine lines (3.33%), tactile roughness (2.86%), global photodamage (0.48%) (Figure 1).

**FIGURE 2:**
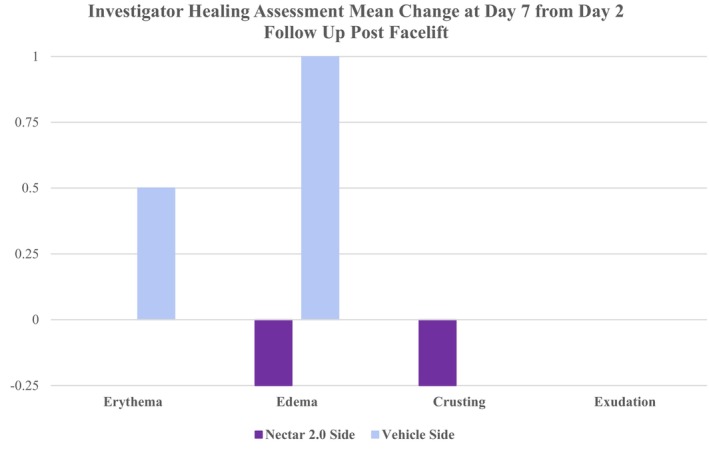
Investigator healing assessment mean change at follow‐up Day 7 EOS from Day 2 post facelift.

Investigator Healing Assessments performed on follow‐up post facelift demonstrated improved healing on the Nectar 2.0 treatment side compared to Vehicle, where the mean change ratings at Day 7 from Day 2 were less on the Nectar 2.0 treatment side compared to Vehicle for the Erythema, Edema, and Crusting parameters (Figure 2).

#### 3.1.2 Subject Assessments and Questionnaires

Subject Self‐Assessment Questionnaire responses reported at Day 7 compared to screening revealed patient perceived improvements for both treatment sides equally for “My skin appears bright” and “My skin looks healthy”, where an improvement (minimum 1 grade/score) was achieved in 80% of patients. Patient perceived improvement was greater for “My skin feels smooth” on the Nectar 2.0 treatment side where 60% of patients achieved improvement at Day 7 compared to Vehicle (40%).

Overall, the percentage of agreement for Nectar 2.0 at baseline post preconditioning was superior to Vehicle for the following Questionnaire statements: The product helps to improve the overall appearance of my skin, I feel the product helps to improve my skin texture, the appearance of fine lines/wrinkles looks reduced, and this is a game‐changing product. All other Questionnaire statements were equal for both products at baseline post preconditioning. At the end of study Day 7, all Questionnaire statements were equal for both products except for the following statements where Vehicle was preferred over the Nectar 2.0 treatment side: The product helps to improve the overall appearance of my skin, my skin looks firmer, my skin looks younger, rejuvenated, and this is a game‐changing product.

End of study survey Subject responses were overall equivalent for both product treatment sides, where 100% of participants indicated they would continue use of and recommend both study topicals. Additionally, 100% of patients also reported overall satisfaction with both topical Test Products, and that they did not cause facial skin dryness, irritation, or redness.

### 3.2 Facelift Surgery and Histology

Biopsy (pre) and facelift surgery skin fragments (post) collected from each side of the face of 4 subjects underwent histological analysis to determine changes following a 4‐week preconditioning period prior to facelift surgery using Nectar 2.0 and Vehicle split‐face twice daily.

**FIGURE 3:**
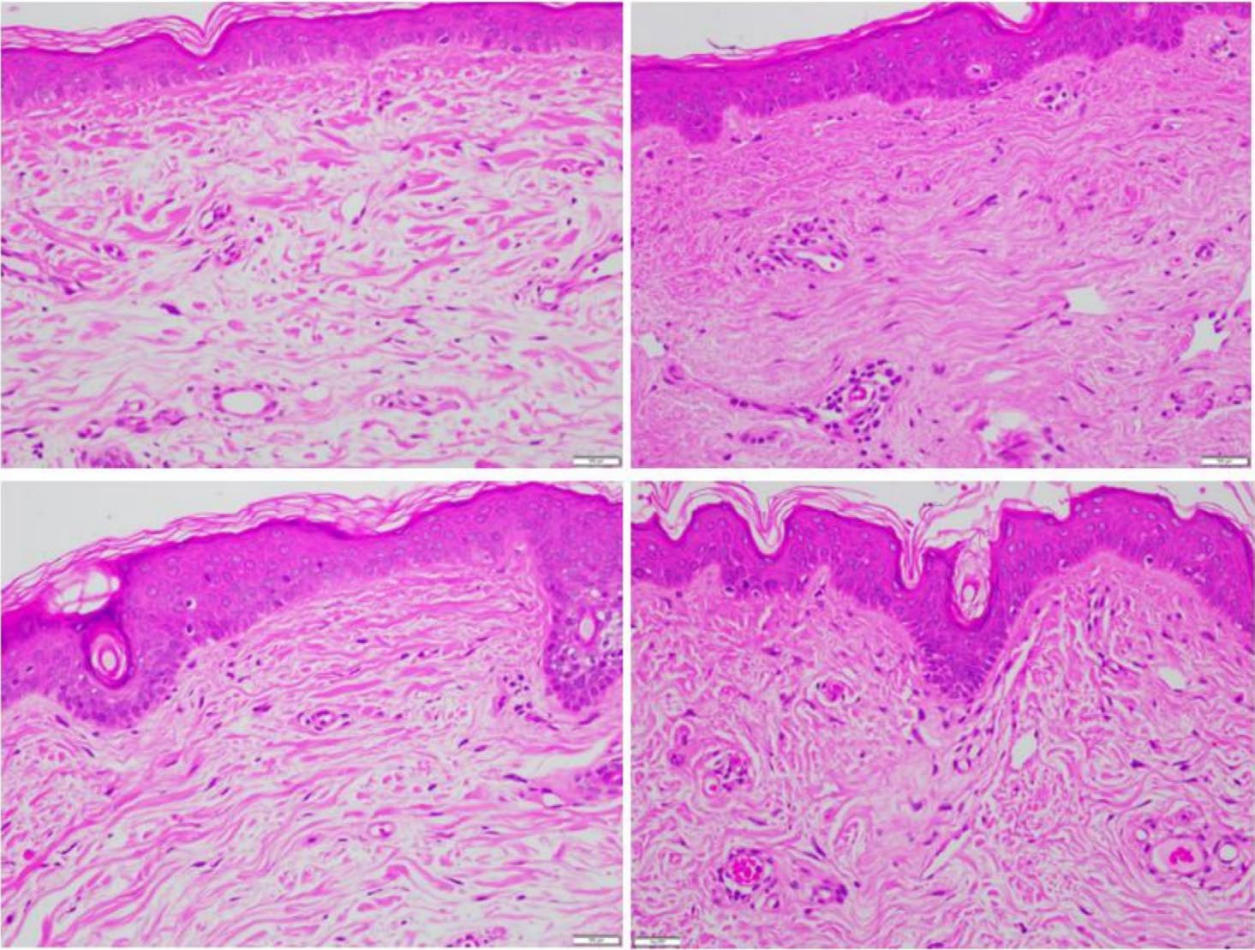
Female, 63 years, Fitzpatrick Skin Type II, H&E staining of biopsies and face lift skin fragments (40× magnification) pre (Left) and post (Right) preconditioning split‐face use of the Nectar 2.0 (Top) and Vehicle (Bottom).

H&E staining of screening biopsies of both sides of the face (Nectar 2.0 and Vehicle) demonstrated the presence of solar elastosis within the dermis, and effacement of rete ridges of the overlying epidermis (Figure 3, Left top and bottom). Face lift skin fragments collected 4 weeks post pre‐conditioning revealed consistent results and changes across all subjects. Representative images are presented demonstrating on Nectar 2.0 side (Figure 3, Right top), a reduction in solar elastosis in the dermis, replaced with tightly packed and well‐organized collagen fibers and more pronounced rete ridges in the epidermis was noted. In contrast, the Vehicle treatment side showed solar elastosis in the dermis and effacement of the rete ridge architecture of the epidermis, similar to the screening biopsy results (Figure 3, bottom).

**FIGURE 4:**
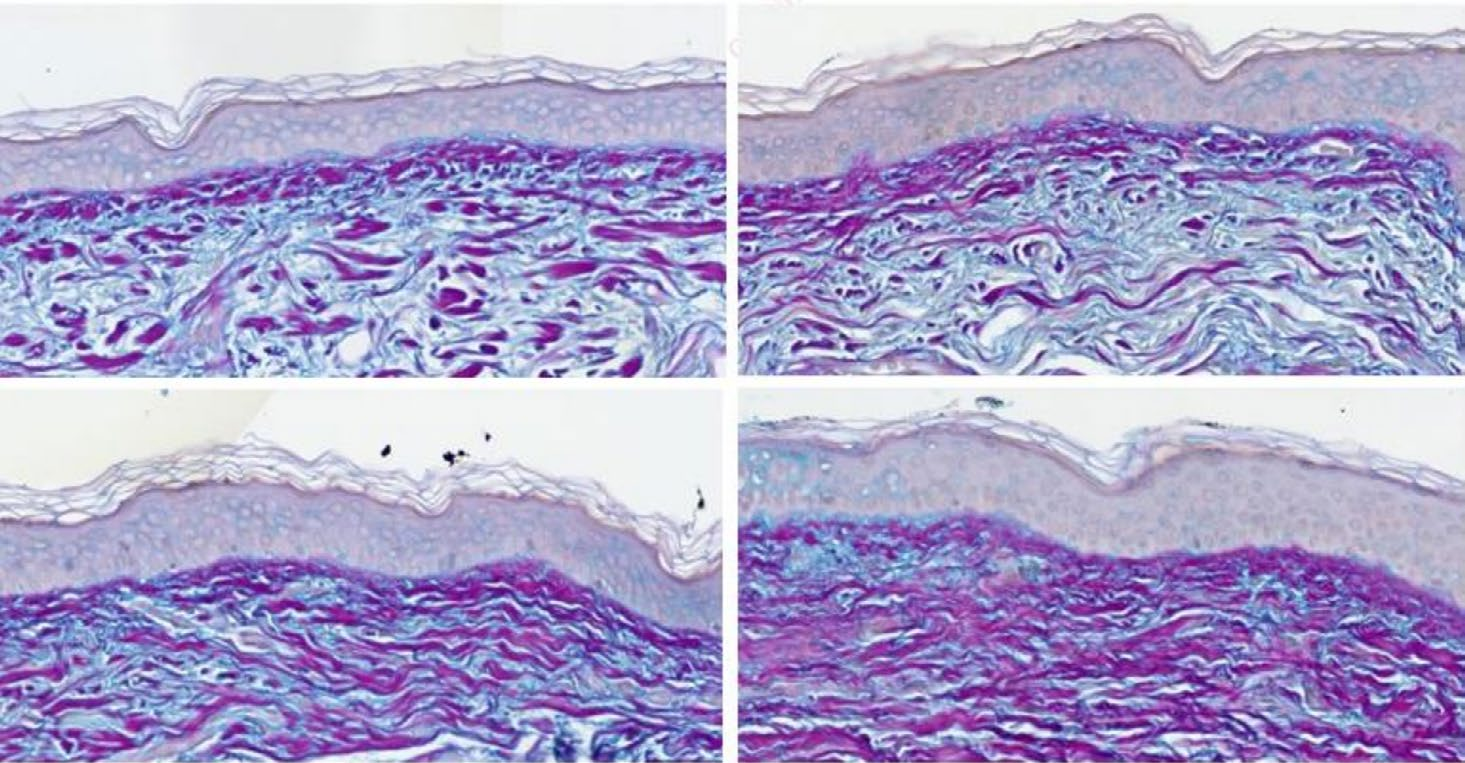
Female, 63 years, Fitzpatrick Skin Type II, Herovici staining of biopsies and face lift skin fragments (40× magnification) pre (Left) and post (Right) preconditioning split‐face use of Nectar 2.0 (Top) and Vehicle (Bottom).

Herovici staining demonstrated increased mucopolysaccharide fibers and new (immature) fine collagen fibers (stained blue) post preconditioning on the Nectar 2.0 treatment side. This in comparison to the Nectar 2.0 screening biopsies (pre), Vehicle screening (pre), and facelift skin fragments (post), which show mature collagen (stained magenta) (Figure 4).

**FIGURE 5:**
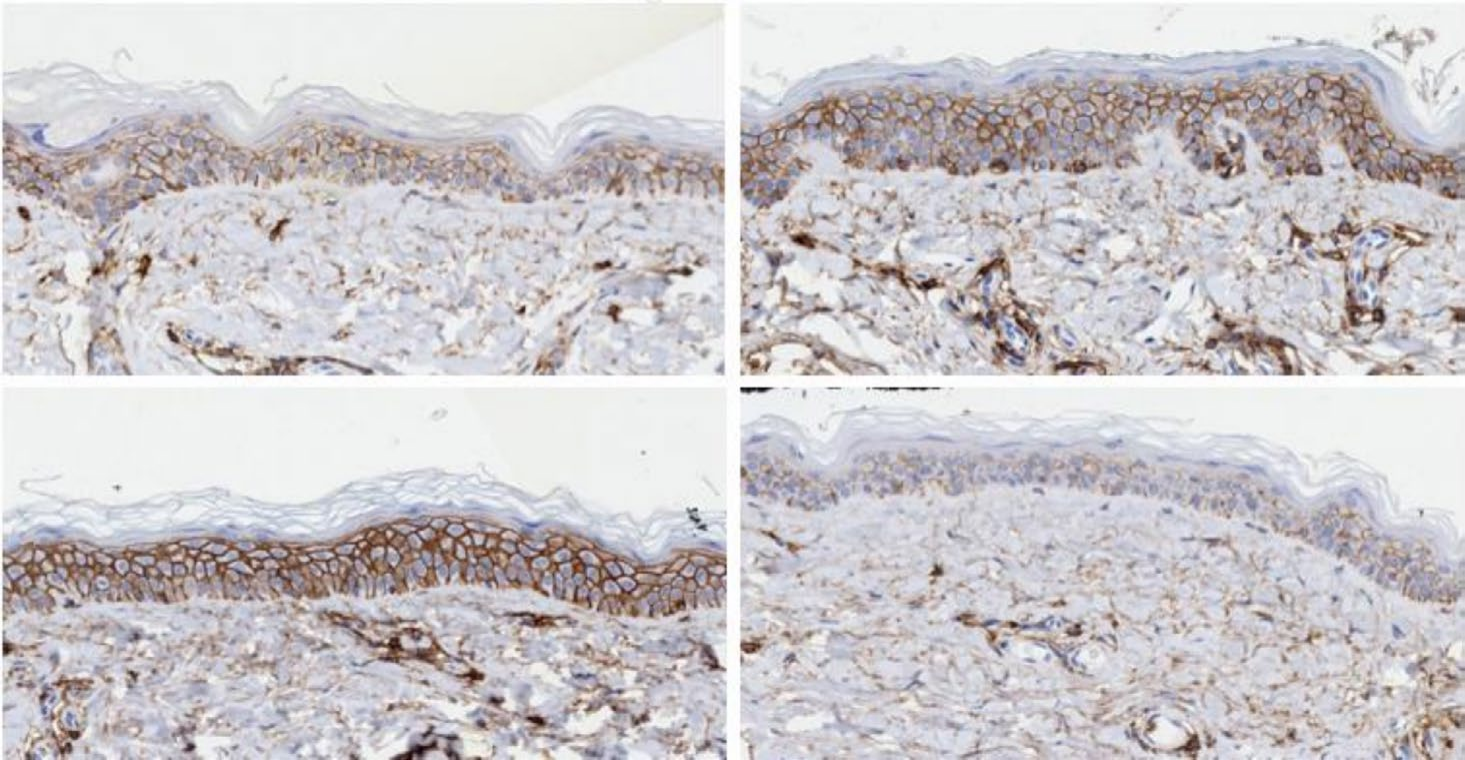
Female, 63 years, Fitzpatrick Skin Type II, CD44 staining of biopsies and face lift skin fragments (40× magnification) pre (Left) and post (Right) preconditioning split‐face use of Nectar 2.0 (Top) and Vehicle (Bottom). Nectar 2.0 achieved a 47.6002% increase in CD44 post preconditioning compared to Vehicle (−51.0489%).

CD44 staining revealed mean change in intensity post 4 weeks of preconditioning, in which an average 65.82% increase was determined for the Nectar 2.0 treatment side compared to a decreased expression for the Vehicle (−33.98%) (Figure 5).

**FIGURE 6:**
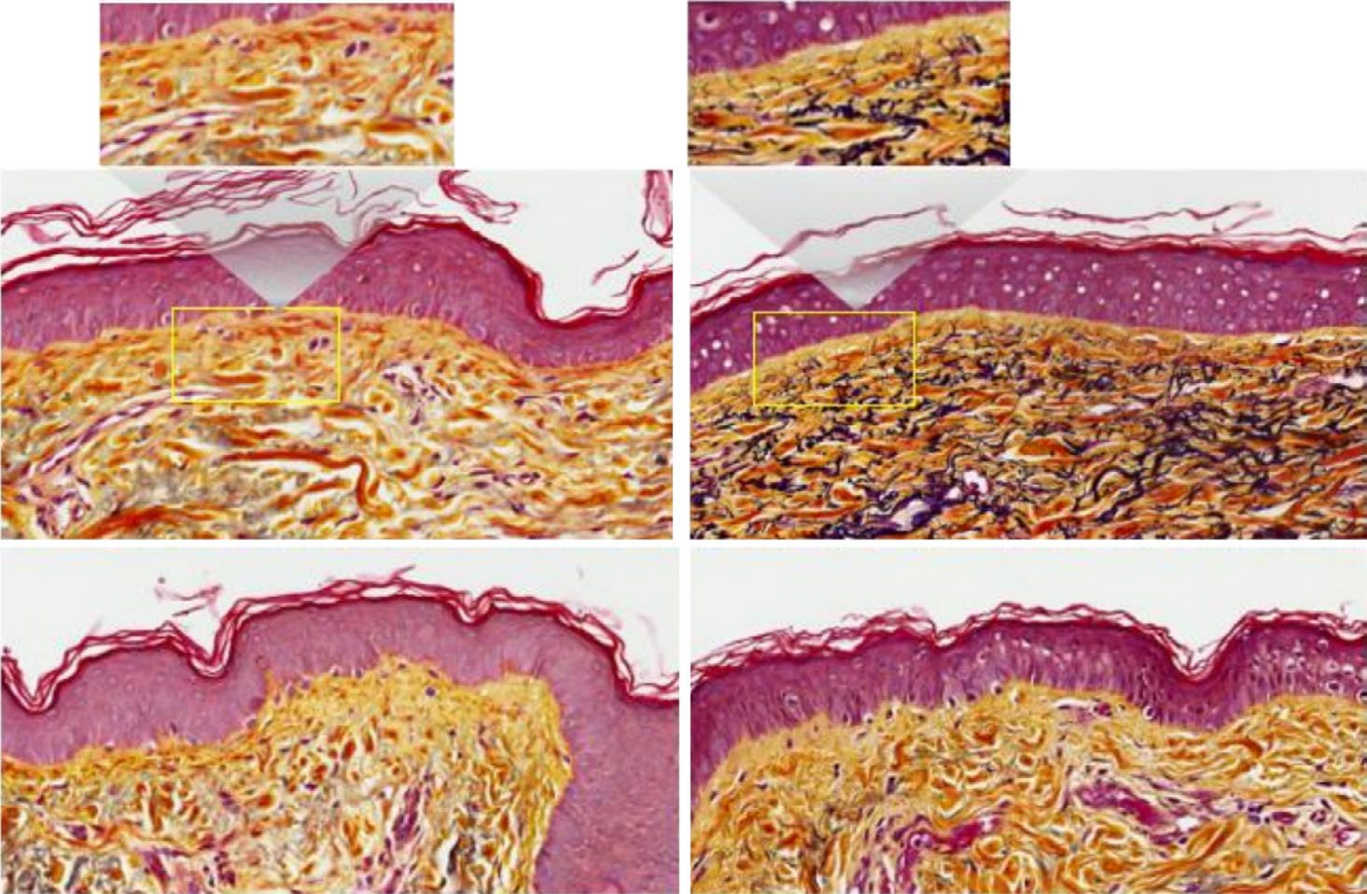
Female, 61 years, Fitzpatrick Skin Type II, Movat stain of biopsies and face lift skin fragments (40× magnification) pre (Left) and post (Right) preconditioning split‐face use of Nectar 2.0 (Top) and Vehicle (Bottom).

Movat staining of screening biopsies demonstrated the presence of minimal foci of short elastin fibers consistent with solar elastosis. This is in contrast to the facelift skin fragments on the Nectar 2.0 side, showing fine elastin fibers in the papillary dermis and improvement in solar elastosis. The Vehicle side is consistent with the screening biopsy side, showing little to no change in the elastin fibers (Figure 6).

### 3.3 Tolerability and Safety

#### 3.3.1 Tolerability Assessments

Tolerability was evaluated on the day of the screening Pre‐Conditioning visit 1b following the first at‐home application of both test products, which demonstrated no reported incidence of any of the parameters (burning, itching, stinging/tingling) evaluated and for either study topical, Nectar 2.0 or Vehicle. Therefore, no substantive differences in tolerability were determined for the two products, which were well tolerated.

#### 3.3.2 Adverse Events

No study test product related adverse events were reported for either study topical product (Nectar 2.0 or Vehicle) in this study.

## 4 Discussion

The primary focus of this study was to document histological changes taking place at a molecular level. We have previously demonstrated that a few weeks of topical intervention can have profound changes in the ECM milieu and those changes translate to improved healing and outcomes [4, 5]. In the cases studied here, the observations were extended to include post‐surgical recovery including erythema, edema, exudate and crusting, and observations on global skin health changes. None of these cases underwent resurfacing procedures in combination with the face lift, so the occurrence of crusting and exudate was anticipated to be minimal (aside from possible crusting of scar), as was observed. However, even following this extremely short period, the investigators did observe some differences in global skin changes, and significant changes in erythema and edema between the 2 sides which appear to reflect the biological/molecular changes seen histologically. These histological changes, the focus of the study, deserve further attention.

In a matter of 4 weeks 100% of patients showed improvements in the ECM components on the Nectar 2.0 treated side vs. the Vehicle side. As a reminder both cleanser and sunscreen were used on both sides, and the study topicals were used split‐face, one side Nectar 2.0, the other side Vehicle. Particularly interesting is the fact that in some of these patients, we were able to observe a significant reversal of solar elastosis, a concept that was refuted in the not‐too‐distant past. Figure 3 Nectar 2.0 (pre) before to after (post) shows sparse ECM with homogenous material creating spaces between the fibers typical of solar elastosis, effacement of the rete ridges and a non‐defined basement membrane dermo‐epidermal junction (DEJ). Following Nectar 2.0 use, we observe a dramatic increase in ECM fiber density, more pronounced rete ridges, and a well‐defined DEJ. This represents a major reversal in skin aging. These changes are confirmed in Figure 4 with Herovici staining, documenting early collagen fiber formation in the form of mucopolysaccharides, a magenta to blue color conversion and an obvious appearance of fine new generating collagen fibers. The increased hyaluronic acid (Figure 5) and elastin generation (Figure 6) all re‐affirm the profound regenerative modulation and remodeling of the ECM observed with pre‐conditioning on the Nectar 2.0 side.

As stated previously, the topical pre‐conditioning concept was introduced 10 years ago and has been validated in a number of studies. This small study was to confirm that an advanced formulation produced ECM remodeling as good as or better than previously observed. This is clearly the case with histological results surpassing those previously documented. More importantly, these molecular changes have clinical benefits as observed by investigators following the post‐surgical healing course and patients' questionnaire responses mirror these positive changes observed.

## 5 Conclusion

A randomized, double‐blinded, split‐face, comparative study was conducted to evaluate the effects of pre‐conditioning with Alastin Regenerating Skin Nectar with TriHex + Technology Serum 2.0 for improving overall skin health compared to the vehicle in subjects undergoing elective facelift surgery. Investigator healing and global skin health assessments showed positive results on the Nectar 2.0 side with distinct differentiation from the vehicle side. This was particularly evident in the histological results with marked remodeling of the ECM being observed in all patients. These changes at a molecular level translate to improved healing and accentuate the concept that a short period of pre‐conditioning can have substantial effects on outcomes.

## Author Contributions

A.D.W.: Developed the science, analysis, paper writing. A.M.: Study investigator, paper writing contribution. F.S.: Study concept and design, paper writing and data analysis. K.R.: Study investigator, paper writing contribution. R.S.: Study investigator, paper writing contribution. T.R.: Study design and management, data analysis, paper writing.

## Funding

This study was supported by Alastin Skincare Inc, a Galderma company.

## Conflicts of Interest

Alan D. Widgerow (Chief Scientific Officer), Faiza Shafiq (Director Clinical Research), Tiffany Robison (Manager, Clinical Research) are all employees of Galderma.

## Data Availability

The data that support the findings of this study are available from the corresponding author upon reasonable request.
